# Supplementary figures and images for: Mediation of the association between stigma and HIV status and fertility intention by fertility desire among heterosexual couples living with HIV in Kunming, China
Source: PLoS One. 2022 Dec 1;17(12):e0278244. doi: 10.1371/journal.pone.0278244 (PMC9714893; doi:10.1371/journal.pone.0278244)

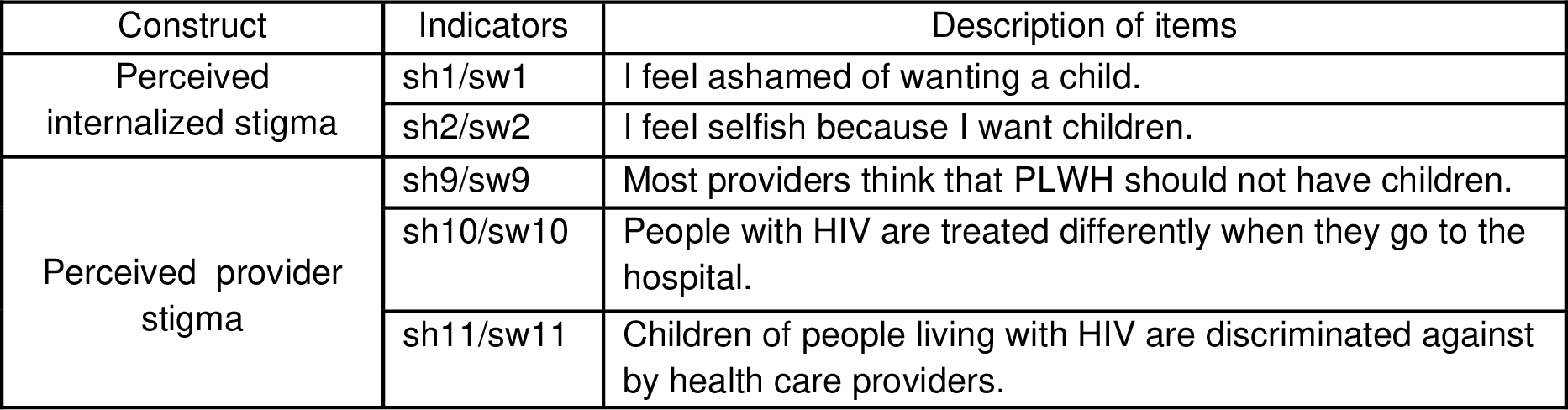

Supplement: S1 Appendix — (TIF) [file pone.0278244.s001.tif]
