# Supplementary material for: Mediation of the association between stigma and HIV status and fertility intention by fertility desire among heterosexual couples living with HIV in Kunming, China
Source: PLoS One. 2022 Dec 1;17(12):e0278244. doi: 10.1371/journal.pone.0278244 (PMC9714893; doi:10.1371/journal.pone.0278244)
Supplement: Attachment — Submitted filename: Response to Editors and Reviewers Comments 20221027.docx [file pone.0278244.s003.docx]

**Manuscript ID: PONE-D-22-21531**

**Response to Reviewers**

Dear Editor and reviewer, `

Thank you for giving us the opportunity to submit a revised draft of the manuscript “**Mediation of the association between stigma and HIV status and fertility intention by fertility desire among heterosexual couples living with HIV in Kunming, China**” for publication in *PLOS ONE*. We appreciate the time and effort that you have dedicated to providing feedback on our manuscript. We are grateful for the insightful comments, which have improved the paper. We have incorporated most of the suggestions, which are highlighted in the manuscript. Below please see our point-by-point response to the reviewers’ comments and concerns.

**Editor’s Comments to the Authors:**

| **Comment** | **Response** |
| --- | --- |
| **Editor** | Author |
| 1. Rebuttal letter: A rebuttal letter that responds to each point raised by the academic editor and reviewer(s). You should upload this letter as a separate file labeled 'Response to Reviewers'. | A rebuttal letter has been prepared on a point-by-point basis and uploaded as a Word document titled “Response to Reviewers” |
| 2. A marked-up copy:  A marked-up copy of your manuscript that highlights changes made to the original version. You should upload this as a separate file labeled 'Revised Manuscript with Track Changes'. | We have prepared a marked-up copy of the manuscript according to the editor’s suggestion. |
| 3. An unmarked version : An unmarked version of your revised paper without tracked changes. You should upload this as a separate file labeled 'Manuscript'. | We have prepared an unmarked version of the revised paper according to the editor’s suggestion. |
| 4. If you would like to make changes to your financial disclosure, please include your updated statement in your cover letter. Guidelines for resubmitting your figure files are available below the reviewer comments at the end of this letter. | (Not applicable) |
| 5. If applicable, we recommend that you deposit your laboratory protocols in protocols.io to enhance the reproducibility of your results. Protocols.io assigns your protocol its own identifier (DOI) so that it can be cited independently in the future. For instructions see: https://journals.plos.org/plosone/s/submission-guidelines#loc-laboratory-protocols. Additionally, PLOS ONE offers an option for publishing peer-reviewed Lab Protocol articles, which describe protocols hosted on protocols.io. Read more information on sharing protocols at https://plos.org/protocols?utm_medium=editorial-email&utm_source=authorletters&utm_campaign=protocols. | (Not applicable) |
| **Journal requirements** |  |
| When submitting your revision, we need you to address these additional requirements.  **1.** Please ensure that your manuscript meets PLOS ONE's style requirements, including those for file naming. The PLOS ONE style templates can be found at  https://journals.plos.org/plosone/s/file?id=wjVg/PLOSOne_formatting_sample_main_body.pdf and  https://journals.plos.org/plosone/s/file?id=ba62/PLOSOne_formatting_sample_title_authors_affiliations.pdf | Thank you. We have prepared the manuscript according to the PLOS ONE template. |
| **2.** Thank you for stating the following financial disclosure:  “Funding was received from the Higher Education Research Promotion and Thailand’s Education Hub for the Southern Region of ASEAN Countries Project Office of the Higher Education Commission (TEH-AC:016/2018)”  Please state what role the funders took in the study. If the funders had no role, please state: "The funders had no role in study design, data collection and analysis, decision to publish, or preparation of the manuscript."  If this statement is not correct you must amend it as needed.  Please include this amended Role of Funder statement in your cover letter; we will change the online submission form on your behalf. | The Role of Funder statement has been added to the Funding sub-section and the cover letter. |
| **3.** In your Data Availability statement, you have not specified where the minimal data set underlying the results described in your manuscript can be found. PLOS defines a study's minimal data set as the underlying data used to reach the conclusions drawn in the manuscript and any additional data required to replicate the reported study findings in their entirety. All PLOS journals require that the minimal data set be made fully available. For more information about our data policy, please see http://journals.plos.org/plosone/s/data-availability.  Upon re-submitting your revised manuscript, please upload your study’s minimal underlying data set as either Supporting Information files or to a stable, public repository and include the relevant URLs, DOIs, or accession numbers within your revised cover letter. For a list of acceptable repositories, please see http://journals.plos.org/plosone/s/data-availability#loc-recommended-repositories. Any potentially identifying patient information must be fully anonymized.  Important: If there are ethical or legal restrictions to sharing your data publicly, please explain these restrictions in detail. Please see our guidelines for more information on what we consider unacceptable restrictions to publicly sharing data: http://journals.plos.org/plosone/s/data-availability#loc-unacceptable-data-access-restrictions. Note that it is not acceptable for the authors to be the sole named individuals responsible for ensuring data access.  We will update your Data Availability statement to reflect the information you provide in your cover letter. | We will upload the study’s minimal underlying data set as a Supporting Information file.  The manuscript now includes the following Data Availability Statement:  "The study's underlying data set is available for download in the Supporting Information section." |
| 4. Your ethics statement should only appear in the Methods section of your manuscript. If your ethics statement is written in any section besides the Methods, please delete it from any other section. | In the revised manuscript, the ethics statement now appears only in the Methods section. |

**Reviewer's Responses to Questions:**

**Comments to the Author**

| **Comment** | **Response** |
| --- | --- |
| Reviewer 1-3 | Author |
| **1. Is the manuscript technically sound, and do the data support the conclusions?**  The manuscript must describe a technically sound piece of scientific research with data that supports the conclusions. Experiments must have been conducted rigorously, with appropriate controls, replication, and sample sizes. The conclusions must be drawn appropriately based on the data presented.  Reviewer #1: Yes  Reviewer #2: Yes  Reviewer #3: Yes | Thank you |
| **2. Has the statistical analysis been performed appropriately and rigorously?**  Reviewer #1: Yes  Reviewer #2: Yes  Reviewer #3: Yes | Thank you |
| **3. Have the authors made all data underlying the findings in their manuscript fully available?**  The PLOS Data policy requires authors to make all data underlying the findings described in their manuscript fully available without restriction, with rare exception (please refer to the Data Availability Statement in the manuscript PDF file). The data should be provided as part of the manuscript or its supporting information, or deposited to a public repository. For example, in addition to summary statistics, the data points behind means, medians and variance measures should be available. If there are restrictions on publicly sharing data—e.g. participant privacy or use of data from a third party—those must be specified.  Reviewer #1: Yes  Reviewer #2: Yes  Reviewer #3: Yes | Thank you |
| **4. Is the manuscript presented in an intelligible fashion and written in standard English?**  PLOS ONE does not copyedit accepted manuscripts, so the language in submitted articles must be clear, correct, and unambiguous. Any typographical or grammatical errors should be corrected at revision, so please note any specific errors here.  Reviewer #1: Yes  Reviewer #2: Yes  Reviewer #3: Yes | Thank you |

**Reviewers' Comments to the Authors:**

***Please use the space provided to explain your answers to the questions above. You may also include additional comments for the author, including concerns about dual publication, research ethics, or publication ethics. (Please upload your review as an attachment if it exceeds 20,000 characters)***

**Reviewer 1**

| **Comment** | **Response** |
| --- | --- |
| Reviewer 1  The manuscript is well written. There are a few minor suggestions: | We thank the reviewer for the comments and suggestions and have attempted to address them. |
| **i)** Overall, there are typos and grammatical errors detected. A professional proof reading is advised. | We thank the reviewer for the comment. The manuscript has been proofread and edited by a native English speaker who has more than 12 years’ experience in medical editing. |
| **ii)** In the methods section the sampling strategy needs to be more clearly described and also add in more details about the study tool (validity etc.) | We have included the following remarks on the sampling strategy and data collection process.  “The study participants included HIV-infected reproductive-age patients attending an ART clinic at the study hospital. The inclusion criteria included: 1) reproductive age (i.e., 20–40 years for females and 20–50 years for males), 2) stable heterosexual relationship where both partners were aware of the HIV status, 3) no child or 1 child, 4) received ART for at least 12 months with undetectable viral loads, 5) no AIDS-defined illness, and 6) living in Yunnan Province. The exclusion criteria were: 1) unable to communicate, 2) too unwell to participate in the interview, and 3) unable to conceive for any reason (e.g., hysterectomy, oophorectomy, medical illness that contraindicated pregnancy, such as complicated heart disease or active autoimmune disease).  One of the investigators worked at the study hospital and prepared a sampling frame based on the list of current patients at the ART clinic in the ART database. The sampling frame data included the patient's clinic-specific identification number, gender, and the date of the next appointment. The investigators used a computer program to randomly sample the patients stratified by sex. On the day of the appointment, investigators approached each patient in person, introduced themselves, assessed the patient's eligibility, provided information about the study, and inquired about the patient's interest in participation. Each participant took approximately 10 minutes to decide whether to participate in the study interview. The investigators then asked for verbal informed consent from the patients before starting the study interview. The patients were also asked for permission to contact their partner to provide information and ask for verbal informed consent. In this study, we refer to one pair of the patient and their partner as a couple.”  Cronbach’s alpha values are shown in Table 2. We have added the average variance extracted values in Table 2 to reflect the discriminant validity of the latent variables. Both outcomes are acceptable.  Revised to:  a) Divergent validity was used to demonstrate that unrelated constructs were actually unrelated by comparing the squared correlations of all latent variables with their average variance extracted values [17].  We have also added the following remarks in the Results section:  In this study, all scales had acceptable levels of Cronbach’s alpha reliability (range = 0.68–0.98) and average variance extracted divergent validity coefficients (0.44–0.96). |
| **iii)** In the discussion section study limitations and justifications need to be explicitly outlined. | We have revised the Discussion section accordingly. We outlined the limitations and justifications following your comments.  Revised to:  **Strengths and limitations**  Our study had a fairly high participation rate from the recruited couples, which potentially reduced the possibility of selection bias. However, a number of limitations should be considered in the interpretation of our study findings. First, the causal pathway in this article was based on an existing theory with a relatively straightforward pathway and a focus on the couple dyad. Findings from a more inductive or inferential approach could have yielded different results. Second, the level of stigma experienced by each participant might have varied over time but our cross-sectional study design precluded the measurement of these variations and reflected mainly the self-reported stigma at the time of the interview. Findings from longitudinal studies could have yielded different results. Third, this study was conducted in Yunnan Province, an area in China with high ethnic diversity and a relatively high level of poverty. The findings of this study may have limited generalizability to couples living with HIV in other settings. |

**Reviewer 2**

| **Comment** | **Response** |
| --- | --- |
| Reviewer 2 | Author |
| **1.**Introduction should be written more clearly and in simple terms rather than just quoting other studies | We have extensively revised the Introduction section according to the reviewer’s comments |
| **2.** Reframe the objectives- should be more clear | We have revised the objectives statement to the following:  “The objectives of this study were to assess the direct influence of stigma and HIV-positive status on fertility intention and the extent that the influence of stigma was mediated by fertility desire among heterosexual HIV-positive husband-wife dyads in Kunming, China.” |
| **3.** When using abbreviations, make sure to expand it when using it for the first time in the manuscript (Ex APIMeM) | We thank the reviewer for the comment and we have made changes accordingly. |
| **4.** Overall Language of the manuscript should be more clear, precise | The manuscript has been revised to improve the clarity. |
| **5.** Key to reading figure should accompany the figure rather than in the middle of the manuscript | We have moved the figure to the end of the manuscript. |
| **6.** What was the amount of time given for giving consent after explaining to the study participants? | We added a sentence in the Methods section to explain the amount of time given to the patients to consider giving consent.  Revised to:  “Each participant was given approximately 10 minutes to decide whether to participate in the study interview.” |
| **7.** What are the other recommendations from the study? | Recommendations are included in the Conclusion section.  Please refer to the following:  “The study findings have implications for family planning counseling in couples living with HIV. The high intra-couple correlation between fertility desire and intention suggests that couples should receive counseling on fertility together, with extensive discussions on concerns regarding HIV-related stigma, potential discrepancies between each partner’s fertility desire and intention, and the influence of one partner on the other.” |

**Reviewer 3**

| **Comment** | **Response** |
| --- | --- |
| Reviewer 3 | Author |
| The manuscript is well written, technically sound and the conclusions were drawn from the results following a robust statistical analysis. The limitations outline further strengthens the work as it clearly aligns the findings with what the study aimed to achieve. I opined that the manuscript needs no further modifications. | We thank the reviewer for the feedback and helpful suggestions throughout this process. |
